# Validation of the Clinical COPD Questionnaire (CCQ) in primary care

**DOI:** 10.1186/1477-7525-7-26

**Published:** 2009-03-25

**Authors:** Björn Ställberg, Mika Nokela, Per-Olof Ehrs, Paul Hjemdal, Eva Wikström Jonsson

**Affiliations:** 1 Department of Public Health and Caring Sciences, Section of Family Medicine and Clinical Epidemiology, Uppsala University, Uppsala, Sweden; 2 Centre for Allergy Research, Karolinska Institutet, SE-171 77 Stockholm, Sweden; 3 Department of Medicine, Clinical Pharmacology Unit, Karolinska University Hospital (Solna), SE-171 76 Stockholm, Sweden; 4 Lung and Allergy Research, Division of Physiology, National Institute of Environmental Medicine, Karolinska Institutet, SE-171 77 Stockholm, Sweden

## Abstract

**Background:**

Patient centred outcomes, such as health status, are important in Chronic Obstructive Pulmonary Disease (COPD). Extensive questionnaires on health status have good measurement properties, but are not suitable for use in primary care. The newly developed, short Clinical COPD Questionnaire, CCQ, was therefore validated against the St George's Respiratory Questionnaire (SGRQ).

**Methods:**

111 patients diagnosed by general practitioners as having COPD completed the questionnaires twice, 2–3 months apart, without systematic changes in treatment. Within this sample of patients with "clinical COPD" a subgroup of patients with spirometry verified COPD was identified. All analyses was performed on both groups.

**Results:**

The mean FEV1 (% predicted) was 58.1% for all patients with clinical COPD and 52.4% in the group with verified COPD (n = 83). Overall correlations between SGRQ and CCQ were strong for all patients with clinical COPD (0.84) and the verified COPD subgroup (0.82). The concordance intra-class correlation between SGRQ and CCQ was 0.91 (p < 0.05). Correlations between CCQ and SGRQ were moderate to good, regardless of COPD severity.

**Conclusion:**

The CCQ is a valid and reliable instrument for assessments of health status on the group level in patients treated for COPD in primary care but its reliability may not be sufficient for the monitoring of individual patients.

## Background

Chronic Obstructive Pulmonary Disease (COPD) is a systemic disease with considerable impact on several dimensions of daily life. The primary aim of treatment is to prevent deterioration of health status/quality of life and to minimize exacerbations which drive quality of life deterioration. Thus, there is a need to evaluate responses to therapy based on these patient related outcomes.

Extensive questionnaires for research purposes provide valuable information, but are time-consuming to fill in and require trained personnel to assist the patient and to calculate the sometimes complicated scoring. These extensive questionnaires have often been validated for group comparisons in patients in chest clinics. Shorter, easy-to-use questionnaires are needed in primary care, as patient visits generally are brief, and nurses and doctors often lack research experience.

The validity of a questionnaire is linked to the context where it is administered. Since patients with mild to moderate COPD [1] are treated at primary health care centres (PHCCs), health status questionnaires for COPD-patients have to be validated in that environment [2]. We therefore performed this study, which is the first validation of the newly developed, brief clinical COPD questionnaire (CCQ) [3] in primary care. St George's Respiratory Questionnaire (SGRQ) [4] was chosen as our gold standard, since it is well validated and frequently used in COPD trials, it is available in a Swedish version, and was used in the original validation of CCQ [5].

## Methods

The study was a prospective multi-centre study in 24 PHCCs located in the Stockholm area. The participating centres had patients with different socioeconomic backgrounds, and doctors and nurses with limited or no experience of research routines. The study was approved by the Ethics Committee of the Karolinska Institutet, Stockholm, Sweden.

### Study population

131 patients diagnosed by general practitioners (GPs) as having COPD were included in the study. Exclusion criteria were age <18 years, malignant disease, severe psychiatric disease, dementia or poor understanding of written Swedish. All participants gave written informed consent. 20 patients were excluded: eight were lost to follow up, seven had incomplete SGRQs, three had spirometric recordings revealing restrictivity, one was an asthmatic included by mistake, and one had a normal spirometry. The excluded patients did not significantly differ from the study population regarding age, sex or smoking habits. The final analysis thus included 111 patients (Table 1).

**Table 1:** Baseline characteristics for the entire study sample with clinical COPD and the subgroup with verified COPD

	**Clinical COPD**	**Verified COPD** (FEV_1_/FVC < 0.70**)
Subjects *n*	111	83
Female (%)	65.8	62.7
Age years, mean, (range)	67.1, (42–85)	67.1, (42–85)
		
Smoking habits (%)		
Non-smokers	2.8	1.2
Ex-smokers	56.9	61.4
Smokers	40.4	34.9
Missing data	1.8	2.4
		
Disease known since, (%)		
< 1 yrs	19.8	10.8
1–5 yrs	37.8	39.8
> 5 yrs	42.3	49.4
		
BMI, mean, (range)	24.4, (17–39)	24.2, (17–39)
FEV_1_/FVC ratio – mean (SD), range	57.8 (14.3), range (26.0–92.6)	52.4 (11.4), range (26.0–69.0)
FEV_1_, % predicted, mean (SD), range	58.1 (20.2), range (14.8–111.5)	52.5 (17.9), range (14.8–102.6)
		
Severity classification, (%) FEV_1 _(post bronch. dil.)		
FEV_1 _≥ 80% predicted	11.0	7.3
50% ≤ FEV_1 _< 80% predicted	52.3	46.3
30% ≤ FEV_1 _< 50% predicted	30.3	37.8
FEV_1 _< 30% predicted	6.4	8.5
Medication visit 1 (visit 2)^#^		
- Only SABA or ipratropium as needed (%)	4.5 (6.5)	6.1 (6.3)
- Ipratropium, tiotropium, LABA or SABA as regular medication (%)	28.2 (26.2)	26.8 (26.6)
- Ipratropium, tiotropium, LABA or SABA *and *ICS as regular medication (%)	50.0 (50.5	54.9 (55.7)
- ICS without any regular brochodilators (%)	3.6 (6.5)	2.4 (5.1)
- No medication (%)	13.6 (10.3)	9.8 (6.3)
- Missing data (n)	1 (4)	1 (4)

*Including four patients with missing data for the ratio FEV_1_/FVC. ** At visit 1.

^#^**Medication during the week preceding the two visits**.

BMI = body mass index, FEV_1 _= forced expiratory volume in one second, SABA= short-acting beta 2-agonist, LABA = long-acting beta 2-agonist, ICS = inhaled corticosteroids.

The COPD-population found in primary care makes up a more heterogeneous population than COPD populations usually included in treatment studies. Correct spirometric evaluations are often lacking in primary care [6]. Nevertheless, spirometry had been performed on all but four patients in our study. Among the 111 patients in our study, 85 were diagnosed as having COPD only, whereas 26 patients were considered to have both COPD and asthma by their treating physician. However, the diagnosis of COPD with or without asthma by the GP did not always meet the spirometric criteria for COPD diagnosis according to Global Initiative for Chronic Obstructive Lung Disease (GOLD)[1]. Nevertheless, we chose to use the GPs diagnosis as inclusion criterion, since this is how patients are diagnosed and treated in primary care. Statistical analyses were performed for the entire study sample with clinical COPD (n = 111) and for the subgroup of patients with spirometry verified COPD (n = 83) which were the major part of the study population. The results from the analyses on the subgroup with verified COPD are reported only if they differed significantly from the results of the primary analyses.

The patients were characterised with regard to age, gender, and pharmacotherapy during the week preceding each visit. Spirometry (FEV1, % of predicted) was performed with ongoing medication according to local routines, but subjected to central evaluation.

### Study design

We compared the 10-item CCQ [3,5,7] with the well validated, extensive SGRQ [4,8,9] on two occasions 10 ± 2 weeks apart without systematic changes in treatment between visits. The time interval was chosen to allow for spontaneous change to occur. If considered needed by the GP, treatment was changed according to local routines after the first visit (Table 1).

The patients completed three questionnaires in their Swedish, self-administered versions in the following order: Short Form-36 Health Survey (SF-36) (Standardised Swedish Version 1.0) [10,11], SGRQ [4,8,9], and finally the authorized Swedish translation of the CCQ provided by the developer [3,5,7]. The questionnaires were filled in before meeting the health professional, i.e. the GP or a nurse. During the meeting The GP or a nurse (according to local routines) estimated if and how the patients' clinical status had changed between the visits.

### Questionnaires

#### SF-36

The SF-36 provides a descriptive measure of generic health related quality of life and is valid for use in COPD [12,13]. The SF-36 was included in the study as a means of characterising the study population. As instructed by the developers, SF-36 was always administered first [10,11]. The SF-36 contains 8 scales that measure physical functioning (10 items), role physical (4 items), bodily pain (2 items), general health (5 items), vitality (4 items), social function (2 items), role emotional (3 items), and mental health (5 items). All scale scores are transformed to range from 0 (worst health) to 100 (best health)[14]. The minimal important difference, MID, for the SF-36 version 1.0 has been reported to range between 3–5 points [15]. SF-36 scores are related to the utilization of healthcare resources by COPD patients [16], and their mortality [17].

#### SGRQ

The SGRQ is a standardized self-administered airways disease-specific questionnaire divided into three subscales: symptoms (eight items), activity (16 items), and impacts (26 items), and 1 overall score. Each score ranges from 0 to 100% (0 = no impairment). The measurement properties of the SGRQ have been found to be satisfactory also in a Swedish population [4]. The recall period in the Swedish version of the SGRQ that was used, is defined as "lately". The minimal important difference, MID, is a score change of ≥ 4 points between occasions [18].

#### CCQ

The CCQ consists of 10 items with an overall score and 3 domains: Symptoms (4 items), Functional state (4 items) and Mental state (2 items). All scores range from 0 to 6; (0 = no impairment). The first validation revealed some weaknesses, such as skewed distributions in functional and mental state domains [5]. The recall period in the Swedish version of the CCQ is defined as the last seven days. The MID for CCQ is 0.41 [19].

#### Clinicians' Global Rating

At visit 2, the GP or COPD-nurse classified changes in the patient's global COPD status as: much worse, worse, stable, better or much better. These ratings were made according to normal clinical routines. There were no instructions given to the GP or nurse as to what to base this rating on. It was left to their discretion to do this according to their professional expertise. The only restrictions made to the ratings were that the patients' individual scores on the QoL questionnaires were blinded for the patient and the GP or nurse at this time.

### Statistical analysis

Non-parametric methods were mainly used, as we did not assume normality of distribution for any variable. For comparison with previous validation studies, however, data in the tables are given as mean ± Standard Deviation (SD) unless otherwise indicated. The software used was SPSS version 12.0.1. (SPSS Inc., Chicago, USA).

Analysis of floor and ceiling effects in all domains in both the CCQ and the SGRQ were made. This was done by calculating the proportion of subjects that had highest possible score and the proportion of subjects that had lowest possible score in each domain.

The closeness of association of CCQ and SGRQ questionnaire data was assessed by Spearman correlation coefficients. We used the following cut-offs: 0 < | r | < 0.3 weak correlation, 0.3 < | r | < 0.7 moderate correlation, | r | > 0.7 strong correlation. The concordance between the instruments was examined with an intra class correlation coefficient.

Measurement properties, intra-class correlation (ICC) and test-retest reliability of the instruments were evaluated using data from a subgroup of stable patients according to SGRQ ratings, which has been defined ± <4 points (the MID) at visit 2.

Test-retest reliability was estimated as the ICC, i.e. the ratio of the between subjects variance and total variance.

Internal consistency, longitudinal and cross-sectional validity were evaluated using data from all patients. Internal consistency was estimated by the Cronbach α (alpha) coefficient [20]. Commonly accepted minimal standards for reliability coefficients are 0.70 for group comparisons and 0.90 for comparisons within individuals [2]. Reliability requirements are higher with individualized use because confidence intervals of the scores are based on the Standard Error of the Mean (SEM), and reliability coefficients <0.9 provide too wide intervals for individual monitoring [2].

To examine cross-sectional validity, we postulated that if the SGRQ and CCQ measure the same construct, they should correlate reasonably well. The a priori expectations were that the total score of SGRQ as well as the symptoms and activity domain scores would correlate strongly with the CCQ total score and with the corresponding domains of CCQ (symptoms and functional state) respectively. For the impacts domain of SGRQ and mental health domain of CCQ, the expectation was that there would be a moderate correlation, since these domains only partially measure the same construct. Only data from the second visit was used.

Longitudinal validity is the ability of the change scores obtained with the investigated instrument to correlate highly, 0 < | r | < 0.3 weak correlation, 0.3 < | r | < 0.7 moderate correlation, | r | > 0.7 strong correlation, with change scores of the criterion/benchmark test SGRQ.

## Results

75% of the 111 patients fulfilled the spirometric COPD criterion of GOLD (FEV1/FVC <70%). 34 patients had very severe or severe COPD, 42 moderate and 7 mild COPD according to the GOLD classification (Table 1). The mean FEV1 (% predicted) in the entire population with clinical COPD was 58.1% (range 14.8–111.5) and in the verified COPD subgroup 52.5%, range (14.8–102.6) (Table 1). FEV1 did not correlate with SGRQ or CCQ scores (data not shown). Table 1 shows baseline characteristics of the patients and medication at both visits. The additional analyses on the subpopulation of patients with verified COPD (FEV1/FVC <70%) at baseline did not in any case significantly differ from the results reported.

**Figure 1:**
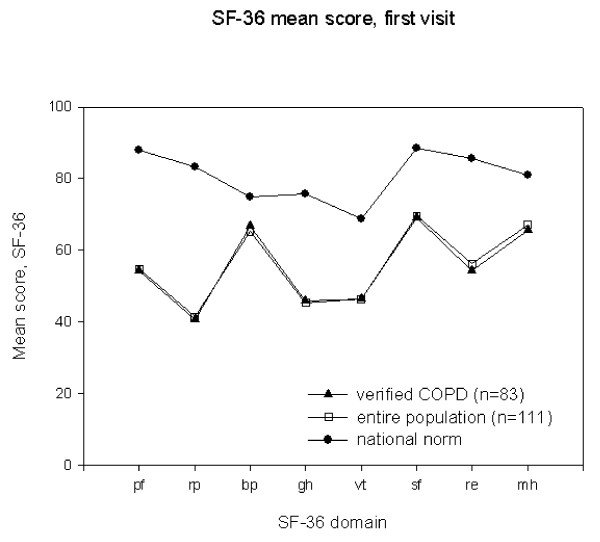
**Baseline SF-36 domain scores for the entire study sample, (n = 111, unfilled squares), COPD verified by spirometry (n = 83, filled triangles)**. National norm data (filled circles) for reference. pf = physical functioning, rp = role-physical, bp = bodily pain, gh = general health, vt = vitality, sf = social functioning, re = role-emotional, mh = mental health.

The entire sample of patients with clinical COPD as well as the subgroup with verified COPD scored lower than the national norm for all SF-36 domains. The SF-36 scores of the subgroup with verified COPD were almost identical with the scores of the entire patient sample with clinical COPD (Figure 1), and this did not change between visits (not shown).

### Comparison of instruments

The total scores of the instruments have completely different scales (Table 2). Nevertheless, the distributions of total scores were similar, and the concordance ICC for the entire population was 0.91 (p < 0.05). The concordance ICC for the Symptom domain was also very good, 0.81 (p < 0.05). Similar results were obtained for the verified COPD subgroup.

**Table 2:** Baseline mean values in each of the instruments for the entire study sample

Domain	SGRQ Mean ± SD	CCQ Mean ± SD	Correlation Spearman	SGRQ Cronbachs alpha	CCQ Cronbachs alpha
Total**	40.91 ± 17.89	2.33 ± 1.03	0.84	0.90	0.84
Symptoms**	47.91 ± 21.92	2.57 ± 1.17	0.70	0.75	0.67
Functional State^#^/Activity*	54.68 ± 23.84	2.03 ± 1.22	0.74	0.84	0.86
Mental State^#^		2.44 ± 1.75			0.82
Impacts*	30.93 ± 18.16			0.82	

** Domain shared by both instruments. * Domain in SGRQ only. # Domain in CCQ only. SGRQ alpha calculated on weighted items. SGRQ = St Georges Respiratory Questionnaire, CCQ = Clinical COPD Questionnaire.

### Floor & Ceiling effects

The total and symptom domain scores of the CCQ were approximately normally distributed. Floor and ceiling effects were negligible (1.8% in the symptoms domain, less in other domains). Distributions in the functional and mental state domains were skewed. In the entire population 4 subjects (3.6%) had optimal functional state scores (0) at visit 1. This increased to 8 subjects (7.2%) at visit 2. Only 1 subject reached the highest possible value at her/his second visit. In the mental state domain, 16 subjects (14.4%) scored optimally (0) at visit 1 and 18 subjects (16.2%) at visit 2; 4 subjects reached the highest possible value at both visits.

The SGRQ did not suffer from floor or ceiling effects. The proportion of subjects that scored at the high or low end were negligible (0 – 3.6%) in all domains and the total score. The pattern was the same for the verified COPD subgroup.

**Figure 2:**
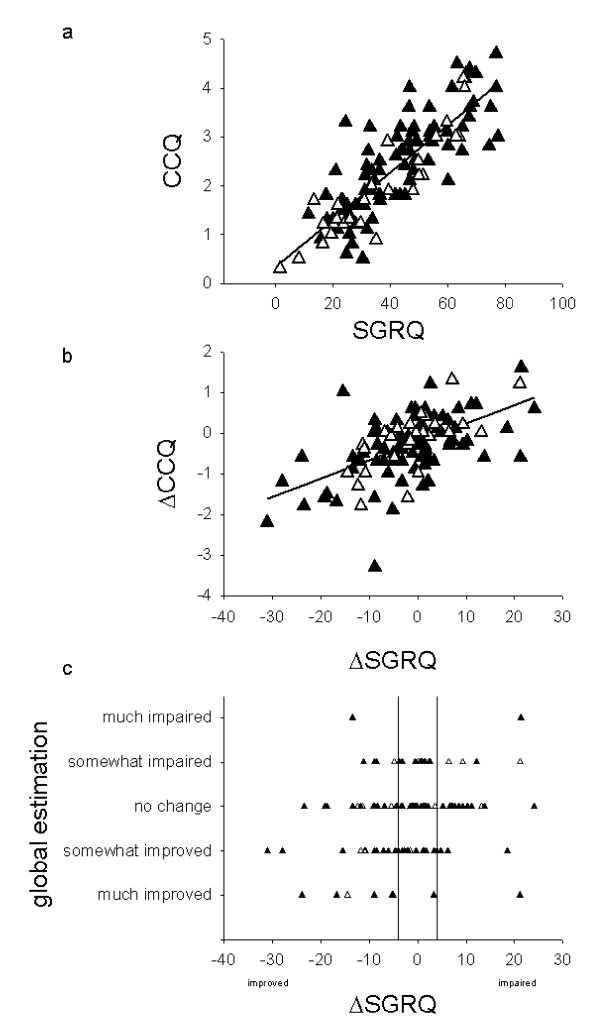
**Relationship between SGRQ scores and other estimations for COPD in the entire study sample (n = 111)**. The group with COPD verified by spirometry (filled triangles), GP diagnosis of COPD not verified by spirometry (empty triangles). a. Scatterplot of SGRQ scores against CCQ scores. Intercept for the regression lines: 0.35: slope 0.048: r^2 ^0.70. b. Change in SGRQ score between visit one and two plotted against change in CCQ score. Intercept for the regression lines: -0.22: slope 0.045: r^2 ^0.32. c. Change in SGRQ scores plotted against GPs estimation of change between visit one and two displays large disagreement between the change in health status as recorded by the patient's SGRQ and the caregiver's estimation of change. The lines represent the minimal important difference (± 4) for SGRQ.

Correlations between SGRQ and CCQ overall scores were strong for the entire population with clinical COPD (0.84; Fig. 2a) and the verified COPD subgroup (0.82, not shown). The Symptoms domains of SGRQ and CCQ correlated moderately (0.70 for clinical COPD). In our study, the internal consistency (Cronbach's alpha) was good, except in the Symptoms domains of both instruments. The Spearman correlation coefficients were good both in patients with FEV1 <50% and >50% of predicted (data not shown).

### Measurement properties

#### Reliability

The reliabilities of the SGRQ and the CCQ were assessed using data from 48 stable patients according to SGRQ scores (23 patients deteriorated and 40 improved and were thus excluded from this analysis). The ICC's were good for both instruments at the overall and domain levels (Table 3). The ICC's for the CCQ were, however, consistently lower than those for SGRQ. The test-retest correlations showed a similar pattern as the ICC's. Similar results were obtained in the verified COPD subgroup (Table 3).

**Table 3:** Reliability of the SGRQ and the CCQ

**Instrument**	ICC^#^ (n = 48)	ICC* (n = 36)
SGRQ		
Total	0.92	0.92
Symptoms	0.80	0.81
Activity	0.84	0.84
Impacts	0.85	0.85
		
CCQ		
Total	0.85	0.85
		
Symptoms	0.74	0.75
Functional state	0.86	0.85
Mental state	0.83	0.83

#Reliability of instruments evaluated on the stable subgroup in the entire study sample with clinical COPD. *Reliability of instruments evaluated on the stable subgroup in the group with verified COPD. ICC (intra class correlation) calculated with a two-way mixed model of consistency. SGRQ = St Georges Respiratory Questionnaire, CCQ = Clinical COPD Questionnaire.

#### Construct validity: longitudinal

Correlations between the CCQ and the SGRQ were moderate to weak for total scores and within domains. The total scores correlated best. Domain correlations were good, especially between functional state in CCQ and Activities in SGRQ (Table 4).

**Table 4:** Cross sectional validity (n = 111)

**Instrument**	**SGRQ**			
	Total	Symptoms	Activity	Impacts
**CCQ**				
Total	0.88	0.77	0.79	0.82
Symptoms	0.77	0.80	0.63	0.70
Functional state	0.82	0.62	0.84	0.73
Mental state	0.64	0.55	0.50	0.63

All correlations significant at the 0.05 level (2-tailed) if not otherwise stated. Summary of sum score correlation coefficents calculated between all domains in both instruments. Calculations based on data from the second visit.

#### Construct validity: cross sectional

Cross-sectional correlations between the SGRQ and the CCQ were fairly good for the entire study sample with clinical COPD (Table 5) and the verified COPD subgroup. The total scores of the two questionnaires correlated best. Some correlations between domains in the SGRQ and CCQ were not significant, but these domains measure very different aspects of health status.

**Table 5:** Longitudinal Validity (n = 111)

**Instrument**	**SGRQ** Total	Symptoms	Impacts	Activity
**CCQ**				
Total	0.52	0.40	0.38	0.31
Symptoms	0.46	0.36	0.36	0.26
Functional				
State	0.44	0.27	0.31	0.33
Mental State	0.30	0.17 n.s	0.18 n.s	0.22

All correlations are significant at the 0.05 level (2-tailed) if not otherwise stated. Summary of change score correlation coefficients calculated between all domains in both instruments. Calculations based on data from both visits from the entire study sample with clinical COPD. SGRQ = St Georges Respiratory Questionnaire, CCQ = Clinical COPD Questionnaire.

Correlations between CCQ and SF-36 indices were poor, with the exception of functional state and the physical index (not shown). A similar pattern was seen in the verified COPD subgroup (not shown).

#### Global ratings

The clinicians' global ratings of improvement/stability/deterioration did not correlate with changes estimated using SGRQ or CCQ (Figure 2c).

## Discussion

Overall, the correlations between CCQ and SGRQ were moderate to good, with a similar pattern to that originally found [5]. The notion that the Functional State domain of CCQ corresponds to the Activity domain of SGRQ was supported. There was also a good correlation between the Impacts and Mental State domains.

Our analysis suggests that CCQ is valid for studies of both mild and moderate to severe COPD in primary care, since equally good correlations were obtained in patients with FEV1 less than or more than 50% of predicted, respectively. The measurement properties of the CCQ were not destroyed by concomitant asthma. The relevance of the present data is supported by the subgroup analysis of patients that at baseline fulfilled spirometric criteria for COPD according to GOLD [1]. Compared to a recent validation of the standardized chronic respiratory questionnaire (CRQ) [21], our results suggest that CCQ has better longitudinal validity but not as good cross sectional validity as the CRQ. However, no direct comparison of the two questionnaires exists. We confirmed 75% of the COPD diagnoses by central examination of spirometries. In a recent Welsh primary care study [6], only 49% of the COPD diagnoses could be confirmed by spirometry. We used the GPs diagnosis of COPD as inclusion criterion in order to validate the CCQ for a COPD population in primary care, where adequate spirometric tests are not common. Of interest, patients who did not fulfill the GOLD requirements for a COPD diagnosis did not worsen the measurement properties of the CCQ (not shown). This is hardly surprising though, since these subjects are probably at least "at risk" subjects, otherwise the GP diagnose of COPD makes no sense.

We found a remarkable lack of agreement between changes in SGRQ health status scores and the clinicians' global ratings, which is in line with previous research [22]. This raises questions as to what the clinicians' rating is based on, and if standardized questionnaires might add value to the primary care consultation.

The SGRQ has properties allowing use at the individual level, but it is extensive and not adapted for everyday use in primary care. The scoring system is complicated, and 7 of our patients were unable to complete the SGRQ acceptably. The reliability coefficient for CCQ was <0.9, suggesting that it may not be sensitive enough for the monitoring of individual patients in ordinary health care [2].

### Limitations

To evaluate the stability of the CCQ, it was tested in the target context under realistic primary care conditions, based on a test-retest design. Studies of test-retest reliability for health-related quality of life instruments have used varying intervals between test administrations. We have found no evidence on which to base the time interval between questionnaire administrations. Short periods will be subject to recall bias and longer periods will eventually lead to changes if the time span is long enough. In order to reflect real life conditions, we chose a relatively long time interval, 10–12 weeks. Then, patients who were expected to remain stable between measurements were selected for the analysis. This was done by using the SGRQ as a GOLD standard. The choice of using the SGRQ as a GOLD standard, might be viewed problematic, but at the time it was (and still is) the best respiratory-specific gold standard available in Swedish. Several reports have shown it to be more responsive to change than generic or preference-based instruments, and it is thus relatively suitable for determinations of temporal stability. This approach may not have been optimal, but we feel that it does not threaten the validity of our result or conclusions.

Another limitation of the study might be the patient selection and whether the severity-distribution of the patients was representative for primary care. Out of the total sample of 111 subjects with clinical COPD, 83 subjects' diagnoses were verified as COPD according to GOLD guidelines. Only four of the 28 subjects who were not classified as having COPD according to GOLD criteria lacked spirometry data.

At baseline, about half of the patients in this study had FEV1 (post bronch. dil.) above 50% of predicted and about half of them below 50% of predicted (table 1). Nine percent had very severe COPD and 38% severe COPD according to the GOLD classification. In a Swedish survey in 2005 with 1096 randomly selected patients with COPD attending primary health care centres in Sweden, 5% had very severe COPD, 26% severe, 44% moderate and 25% mild COPD according to pulmonary function tests (unpublished data). Considering these figures, we assume that the patient group in our study is probably representative for the COPD population in primary care in Sweden.

## Conclusion

In conclusion, our results indicate that the CCQ has good measurement properties for studies of health status at the group level, whereas its reliability may not be sufficient for the monitoring of individual patients. The CCQ is easy to score, and allows data to be quickly collected and processed, and is thus suitable for use in every day practice for clinical trials or quality of care monitoring.

## Competing interests

The authors declare that they have no competing interests.

## Authors' contributions

BS has made substantial contributions to conception, design, acquisition of data, analysis and interpretation of data; he has also been involved in drafting the manuscript and revising it critically. MN has been involved in the acquisition, analysis and interpretation of data, as well as in drafting the manuscript and revising it. POE contributed to the conception, design, acquisition and interpretation of data. PH and EWJ both made important contributions to the conception, design, analysis and interpretation of data, and revised the manuscript critically.
